# Identification of *Abies sibirica* L. Polyprenols and Characterisation of Polyprenol-Containing Liposomes

**DOI:** 10.3390/molecules25081801

**Published:** 2020-04-14

**Authors:** Ilona Vanaga, Jerzy Gubernator, Ilva Nakurte, Ugis Kletnieks, Ruta Muceniece, Baiba Jansone

**Affiliations:** 1 Department of Pharmacology, Faculty of Medicine, University of Latvia, Jelgavas str. 3, LV-1004 Riga, Latvia; ruta.muceniece@lu.lv (R.M.); baiba.jansone@lu.lv (B.J.); 2 LTD “Silv EXPO”, Alberta str. 12-2, LV-1010 Riga, Latvia; 3 JSC “Biolat”, Rigas str. 111, LV-2169 Salaspils, Latvia; 4 Faculty of Biotechnology, University of Wroclaw, Joliot Currie 14A, 51-383 Wrocław, Poland; jerzy.gubernator@uwr.edu.pl; 5 Institute for Environmental Solutions, „Lidlauks”, Priekulu parish, LV- 4101 Priekulu county, Latvia; 6 Department of Physical Chemistry, Faculty of Chemistry, University of Latvia, Jelgavas str. 1, LV-1004 Riga, Latvia

**Keywords:** polyprenols, *Abies sibirica* L., liposome, physicochemical properties, HPLC

## Abstract

The needles of conifer trees are one of the richest sources of natural polyprenols. Polyprenol homologs from *Abies sibirica* L. lipophilic 80% purified extract were analyzed and quantified. In total, 10 peaks (Prenol-11 to Prenol-20) were observed in the ultra-high-performance liquid chromatography–diode array detector (UHPLC-DAD) chromatogram of Siberian fir with the most abundant compound being Prenol-15 (relative amount 37.23 + 0.56% of the total polyprenol yield). *Abies sibirica* L. polyprenol solubility and incorporation efficiency into liposomes were studied in various commercially available lecithin mixtures (Phosal IP40, Phosal 75SA, and Lipoid P45). The resulting multilamellar polyprenol liposomes were morphologically characterized by Light and Transmission Electron Microscopy, and the liposome size was discovered to be polymodal with the main peak at 1360 nm (90% of the volume). As polyprenols are fully soluble only in lipids, a liposomal formulation based upon co-solubilization and a modified ethanol injection method of polyprenols into the ethanol-phospholipid system was developed for the entrapment and delivery of polyprenols for potential commercial applications in food supplement and cosmetic industries.

## 1. Introduction

Polyprenols are a well-known class of natural substances that have mostly been studied for their anti-inflammatory [1], hepatoprotective [2], and immunomodulating functions [3]. Recently, polyprenols were also tested in muscle strength and coordination experiments in combination with widely used drugs statins, reducing their side effects *in vivo* and in patients [4,5]. Polyprenols are hydrophobic molecules of natural long-chain isoprenoid alcohols. Their general formula is H-(C5H8)n-OH, wherein n represents the number of isoprene units [6]. Polyprenols extracted from different plants and also animal sources vary in chain lengths of their homologs and geometrical configuration [7]. Popular plant sources for polyprenol extraction on a commercial scale are conifer biomass such as *Abies sibirica* L., *Picea abies* L., *Pinus sibirica* L., *Pinus sylvestris* L. [8] and *Ginkgo biloba* L. [9,10]. Previously, the chemical composition of *Pinaceae* polyprenols has been described in their phosphorylated [11,12,13] and acetate forms [14]. Moreover, various *P. abies* species have been analyzed and described chromatographically [15], but an in depth description of *Abies sibirica* L. polyprenol homologs has not been presented.

As free substances, polyprenols have limited bioavailability due to high hydrophobicity and limited emulsification in the digestive tract [16]. Due to these limitations of polyprenols, a liposome is an accommodating form of polyprenol delivery. Liposomes are spherical structures composed of phospholipids or other amphiphiles having the structure of the lipid bilayer [17]. A liposome can be considered a universal drug carrier since it can accommodate hydrophilic (in liposome’s water compartment), hydrophobic (in the liposome’s bilayer), and lipophilic substances (both in the liposome’s bilayer and in liposomal water compartments depending on the physicochemical properties) [18]. These rare liposomal characteristics enable their use in the pharmaceutical, cosmetic, and diet supplement industry [19]. Additionally, liposomes are a versatile system used in biophysical and molecular biology studies [20].

Liposomes can be prepared from various components, including the glycerol derivatives of natural phospholipids, e.g., phosphatidylcholine, phosphatidylethanolamine, phosphatidylserine, phosphatidylglycerol, or the derivatives of sphingosine – sphingomyelin [21]. Liposomes can also be formed from natural ceramides, glycolipids, and other lipids having the ability to form lipid bilayers in aqueous media [22]. Liposomes can be divided into multilamellar, small unilamellar, and large unilamellar vesicles being in size from about 30 nm for small unilamellar vesicles to several microns for multilamellar vesicles [23]. In order to prolong the stability of liposomes, some semi-natural phospholipids can be used in their production, that are able to circulate for long periods of time in the body [24]. Some types of liposomes (niosomes) can be prepared from surfactants having the ability to form the lipid bilayer alone or together with cholesterol [25,26].

The aim of this study was to develop a liposomal delivery system for conifer polyprenols for food supplements and also cosmetic industry applications. In order to ensure the physical stability and absorption of this formulation via oral and dermal routes, *Abies sibirica* L. polyprenol quantitative and qualitative chromatographic profile was described. Polyprenol solubility study and incorporation method into liposomes and thereof physical properties (particle size, polydispersity, encapsulation efficiency, and morphological analysis) were studied as well.

## 2. Results

### 2.1. The Identification of Siberian fir (Abies sibirica L.) Polyprenol Homologs

**Figure 1:**
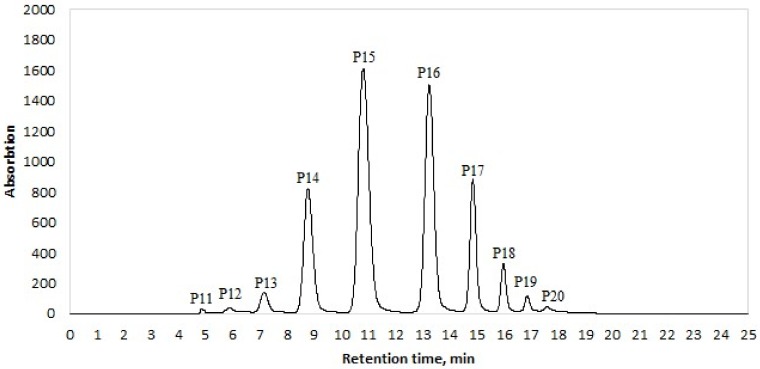
Ultra-high-performance liquid chromatography–diode array detector (UHPLC-DAD) chromatogram of extracted polyprenols from Siberian fir *(Abies sibirica* L.). UHPLC conditions: Agilent Extend C18 2.1 × 50 mm, 1.7 μm column; mobile phase: A (methanol) and B (isopropanol) under gradient program; flow rate 0.22 mL·min^−1^; column temperature 40 °C; detection wavelength 210 nm.

Identification of the separated polyprenol homologs from Siberian fir (*Abies sibirica* L.) extract was mainly based on UV spectral data with the aid of chemical standards and the high-resolution mass spectrometry (HRMS) search for [M + Na]^+^ polyprenol ions, using extracted ion mass chromatograms, and also taking into account the data provided by the external standard (Table 1). High-resolution mass spectra for all polyprenol homologs are available in Supplementary Materials (Figure S1). Experimentally obtained HRMS spectra of separated P11 to P20 homologs approved the calculated values (P11 - C55H90O (M + Na)^+^ calc 789.6867, (M + Na)^+^ found 789.6867, Δ = 0.0017; P12 - C60H98O (M + Na)^+^ calc 857.7503, (M + Na)^+^ found 857.7503, Δ = 0.0007; P13 - C65H106O (M + Na)^+^ calc 925.8134, (M + Na)^+^ found 925.8134, Δ = 0.0002; P14 - C70H114O (M + Na)^+^ calc 993.8762, (M + Na)^+^ found 993.8779, Δ = 0.0017; P15 - C75H122O (M + Na)^+^ calc 1061.9388, (M + Na)^+^ found 1061.9372, Δ = 0.0016; P16 - C80H130O (M + Na)^+^ calc 1130.0014, (M + Na)^+^ found 1130.0017, Δ = 0.0003; P17 - C85H138O (M + Na)^+^ calc 1198.0640, (M + Na)^+^ found 1198.0650, Δ = 0.0010; P18 - C90H146O (M + Na)^+^ calc 1266.1274, (M + Na)^+^ found 1266.1274, Δ = 0.0008; P19 - C95H154O (M + Na)^+^ calc 1334.1893, (M + Na)^+^ found 1334.1893, Δ = 0.0001; P20 - C100H162O (M + Na)^+^ calc 1402.2577, (M + Na)^+^ found 1402.2577, Δ = 0.0059). Figure 1 shows that ten peaks in total were observed in UHPLC-DAD chromatogram of Siberian fir (*Abies sibirica* L.) extract. All separated peaks were identified as polyprenols. The most abundant compound was found to be P15 with its relative amount 37.23 + 0.56% of the total polyprenol yield.

### 2.2. The Solubility of Polyprenols in Ethanol and Ethanolic Solution of Lecithin

**Figure 2:**
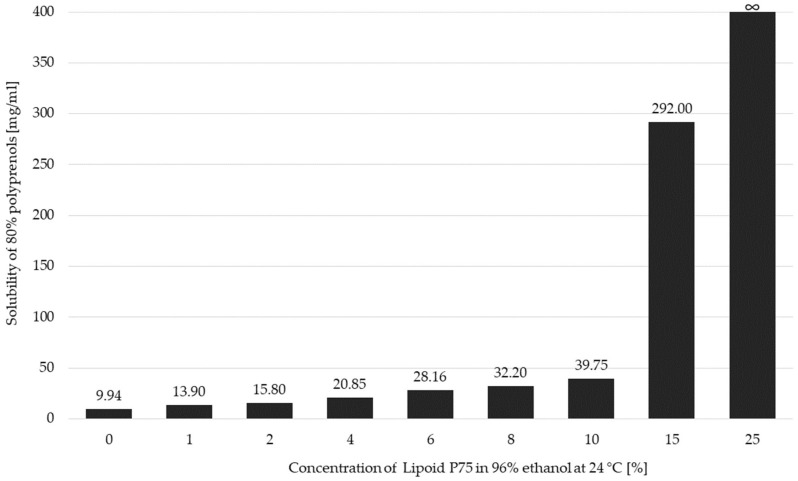
Influence of the lecithin (Lipoid P75) concentration in ethanol on the solubility of *Abies sibirica* L. spruce polyprenols.

A new method based upon co-solubilization and the modified ethanol injection method of polyprenols in the ethanol-phospholipid system was developed. Polyprenols have limited solubility in ethanol, but it increases with the content of phospholipids in the mixture. Figure 2 shows the increase of solubility of conifer polyprenols in ethanol as a function of the increased concentration of the 75% soya phosphatidylcholine with practically unlimited solubility of 80% polyprenols being reached at 25% of the Lipoid P75 mixture in 96% ethanol at 24 °C. The co-solubility facilitates achieving high liposome concentration and low amounts of alcohol in the final product.

**Figure 3:**
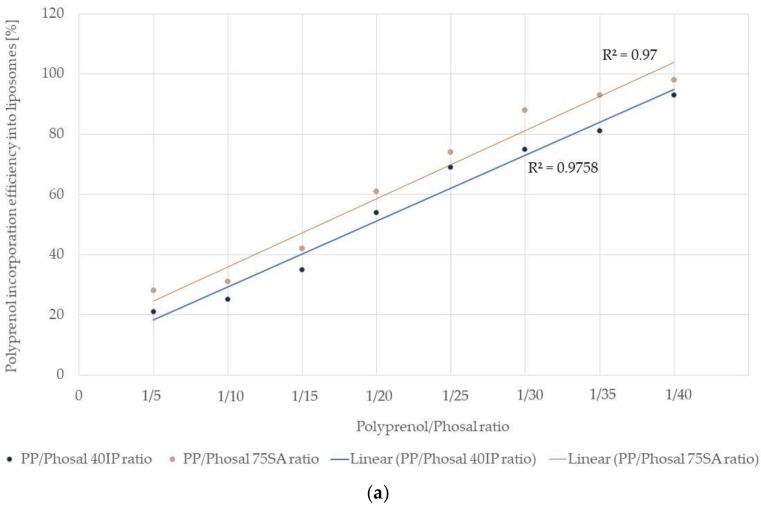

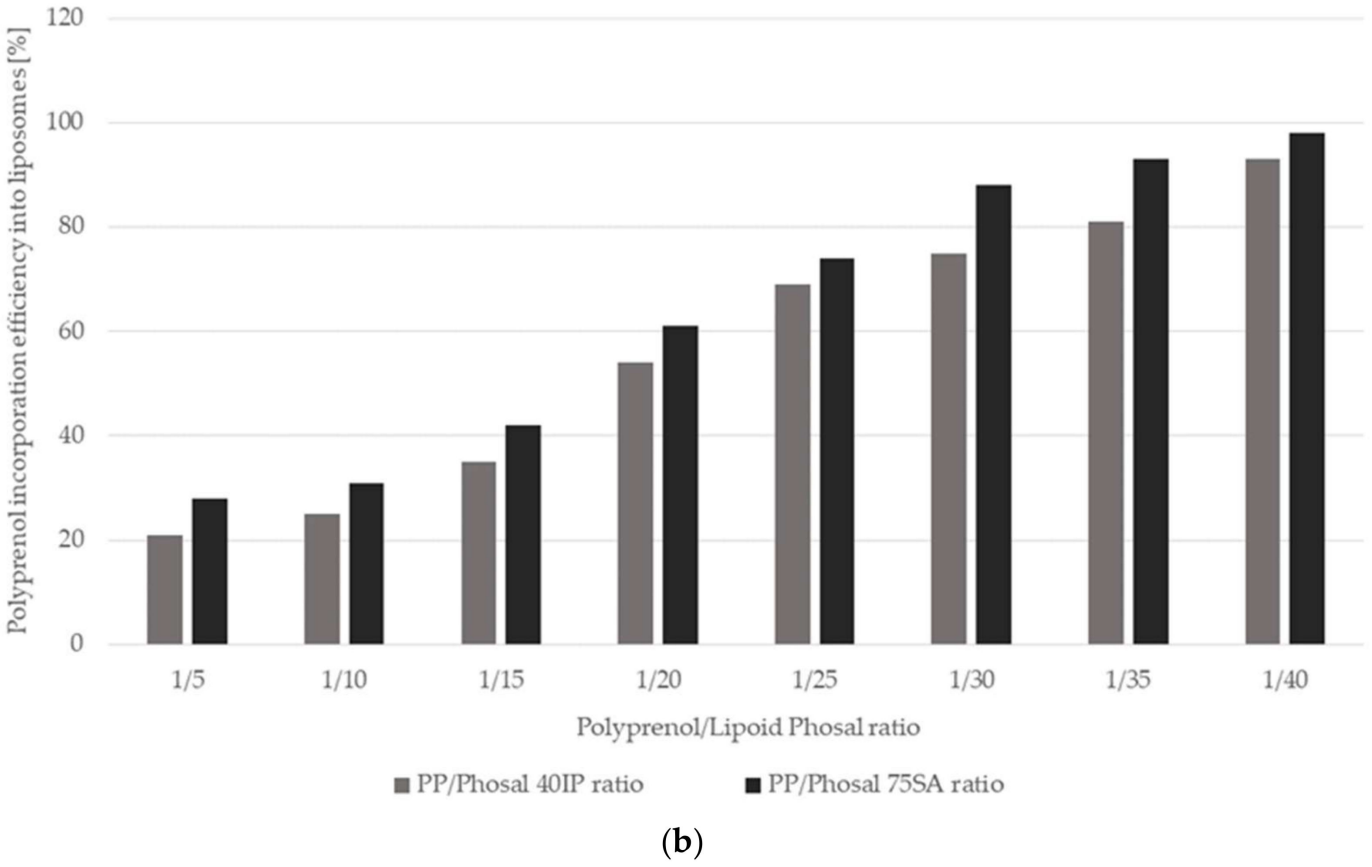
Influence of the polyprenol/lecithin (Phosal IP40 and Phosal 75SA) ratio on the *Abies sibirica* L. spruce polyprenol incorporation efficiency into liposomes: (**a**) scatter diagram and (**b**) column chart.

As shown in Figure 2, for the full dissolution of lecithin in ethanol, at least 40% of phosphatidylcholine must be present in the raw material; therefore, the phosphatidylcholine content in the used material must be in the range from 40% to 100%. The phospholipid/polyprenol ratio must be higher than 1:15 in order to achieve stable liposomal formulation. A negative correlation was found between the incorporation efficiency of 80% polyprenols and their dissolved ratio in the Phosal mixtures with the highest efficiency being reached at a ratio of 1/40 for both polyprenols/Phosal 40IP and polyprenols/Phosal 75SA mixtures (Figure 3). When the polyprenol/phospholipid ratio is lower, polyprenol emulsion and phospholipid liposomes are formed separately instead of polyprenol liposomes (Table 2).

### 2.3. Morphological Observations of Polyprenol-Loaded Liposomes

**Figure 4:**
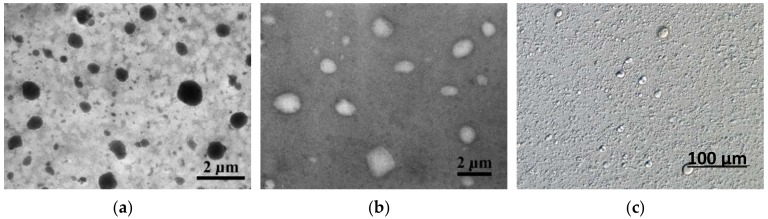
Images of polyprenol/Phosal 40 IP liposomes (1:17 *w*/*w*) by Transmission Electron Microscope (TEM): (**a**) positive staining; (**b**) negative staining; and (**c**) light microscope.

**Figure 5:**
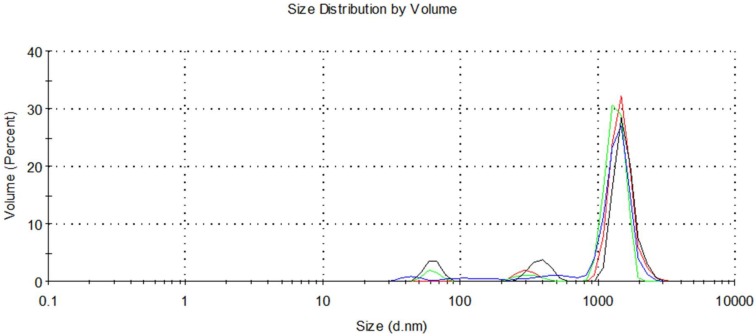
Size distribution of polyprenol/Phosal 40 IP liposomes (1:17 *w*/*w*).

Polyprenol liposomes were assessed microscopically, and Figure 4 displays typical multilamellar liposomes. As seen in Figure 5, liposome size was discovered to be polymodal with the main peak at about 1360 nm (90% of the volume) and two smaller populations at size 307 nm (~5%) and 62 nm (~5%). The biggest particles are in the size of about 4–5 μm, whereas the smaller ones are about 25 nm in size. According to the Zetasizer quality report, some larger than 5 μm particles were also present in the sample, but they are only a very small fraction of the total liposomal mass.

## 3. Discussion

Polyprenols have been arousing interest already since the 1960s [27]. Nowadays, we have much more information not only on the synthetic pathways of polyprenol and dolichol (α-saturated isoprenoids) formation [28] but also further scientific questions are being asked, whether polyprenols and dolichols are super-lipids [29] and to what degree they can be applied in prophylaxis and treatment of different diseases [30]. So far, polyprenols have been extracted from a plethora of various plants [7,31,32], but many novel synthesized derivatives have been described as well [33]. Analytically speaking, the most thoroughly studied isoprenoid structures seem to be those extracted from *Ginkgo biloba* [34] plausibly due to plant’s high popularity in the Eastern traditional medicine. Apart from that, there are several studies that examine polyisoprenoid-containing bilayer lipid membrane models [35,36,37] and liposomal formulations [38]. All the accumulated research notwithstanding, there are still only a few commercial polyprenol formulations such as Ropren^®^ [39], Fortepren^®^ [3], and antiviral polyprenol aerosol [40] available on the market today, and thus we wished to explore the potential of such polyprenol preparations further.

In our study, we focused on the characterization of *Abies sibirica* L. derived polyprenols with prevalent isoprenoid alcohol chain lengths of C_55_–C_100_ (isoprene unit count 11–20), which correspond to the general description of conifer polyprenol extracts [7,41]. The main intent was to develop a commercially viable polyprenol delivery system that could be used for food supplement and cosmetic applications. For this purpose, liposomes seemed to be the best carrier candidate for polyprenols, since they possess emulsifying properties and can accommodate polyprenol molecules as a part of the bilayer [42,43]. The customary industrial methods of liposome preparation used in diet supplements include ethanol injection, fluidization, sonication, classic homogenization, or high-pressure homogenization methods [44].

Liposomes are very convenient products often used in diet supplements, especially to encapsulate hydrophobic compounds in order to increase their bioavailability [45]. Despite that, in our study, we came across some drawbacks, e.g., polyprenols have limited ability to co-form liposomal bilayer due to mismatched length of their hydrophobic chains, which are sometimes much longer than the bilayer thickness. This aspect leads to liposome destabilization, when increased amounts of these molecules are incorporated into the lipid bilayer [46]. Therefore, a low polyprenol and liposomal lipid weight ratio must be used in order to achieve a stable liposomal suspension. Another limitation corresponds to the low solubility of polyprenols in the organic solvents conventionally used in the diet supplement industry [47]. For example, the solubility in glycerol or propylene glycol is almost negligible. The solubility of polyprenols in ethanol is only 10 mg/mL, whereas at least ten times higher concentration is required to prepare a liposomal supplement, which is a problem if one wishes to produce a formulation with a low content of ethanol.

The most difficult step in the liposomal polyprenol production comprised the preparation of the lipid film or lipid mass, which had to entrap a desired portion of the polyprenols. In the case of hydrophobic molecules like polyprenols, curcumin, ubiquinone, quercetin, etc. a complete dissolution in organic solvent must take place in order to achieve high incorporation efficiency into the liposome [48]. With the purpose of producing a polyprenol formulation containing almost no alcohol, another lipid system had to be used - the so-called fluid lecithins. The fluid lecithins are prepared by milling of the dry lecithin of various sources with triglycerides containing medium chain length fatty acids with an addition of a small amount of alcohol. There are several fluid lecithins available on the market, but most of them have bakery purity grades at most, and others are composed of genetically modified lipids (for instance, Lipoid Phosal 75 SA), which are forbidden to be used in any supplements in the European Union. We found that the most appropriate product for our design was Lipoid Phosal 40 IP, which contained at least 40% of the phosphatidylcholine and at least 30% of other phospholipids. Polyprenols then could be mixed with fluid lecithin to form a solution, which yielded multilamellar liposomes upon hydration. The resulting liposomes could also be further homogenized by sonication or high-pressure homogenization to produce liposomes of the desired size.

The Transmission Electron Microscopy (TEM) findings of the soybean phosphatidylcholine multilamellar liposomes that entrap polyprenols indicate vesicles of a fairly regular, spherical form and according to literature, polyprenols are most likely incorporated into the phospholipid bilayer [49]. Also, as discussed in [42], polyprenol interactions with the lipid bilayer influence the properties of the liposome and can alter its permeability and fluidity, depending on the content of the entrapped polyisoprenoid alcohols. Apart from that, important aspects for potential delivery and commercial applications are also the significant protection against chemical degradation and the increased effectiveness and cellular uptake of the encapsulated material, which the liposomal form provides, thereby extending the product’s shelf life and bioavailability [50].

## 4. Experimental Section

### 4.1. Materials and Instruments

Siberian fir *(Abies sibirica* L.) polyprenols (purity ~80%) were acquired from JSC “Biolat” (Salaspils, Latvia), where they had been extracted according to the procedure described in the patent of the Russian Federation No. RU2003123562A [51] and [52]. Phospholipid mixtures, Phosal 40 IP and Lipoid P75 (contain at least 25–75% of soybean phosphatidylcholine), were purchased from “Lipoid” GmbH (Ludwigshafen, Germany). External quantitative standard Pren-14 and a mixture of polyprenols (Standard P14-P18 mix *Pinus sylvestris* L. Batch 08-10 JTC; Code 89-5170) was acquired from the Institute of Biochemistry and Biophysics (Polish Academy of Sciences, Warsaw, Poland). The identity and structure of polyprenol homologs were confirmed by high-resolution time-of-flight mass spectrometry (Agilent 6230 TOF LC/MS; Agilent Technologies Deutschland GmbH, Waldbronn, Germany) using positive electrospray ionization (ESI+). Peak detection and data acquisition were provided by the MassHunter Qualitative Analyses B.05.00 data processing system. The multilamellar liposomes were examined under high-grade optical Nikon Eclipse 90i microscope (Nikon Instruments Co., Tokyo, Japan) equipped with Nomarski contrast. The Transmission Electron Microscopy (Tesla BS 540 JEOL 100, Tesla, Brno, Czechoslovakia) was used to observe the microstructure and morphology of polyprenol containing multilamellar liposomes using a negative-staining method. All chemicals employed for liquid chromatographic studies were of HPLC (high-performance liquid chromatography) grade.

### 4.2. Sample Clean-up for UHPLC Analysis

The obtained 70–85% *Abies sibirica* L. polyprenol extract was applied to column chromatography on silica gel and eluted with petroleum ether (PE)/ethyl acetate (EA) (93:7) mixture. The weighted sample amount to silica gel was 1:25 to 1:30. The elution rate was approximately one drop/second. PP control was performed via Silufol thin-layer chromatography (TLC). As a result, acquired *Abies sibirica* L. polyprenols were of 95–98% purity with a yield of 0.7–0.8% (*w*/*w*) The resulting product was low-viscosity pale-yellow oil insoluble in water and methanol, but well soluble in hexane, acetone and other non-polar organic solvents.

### 4.3. Sample Preparation for UHPLC Analysis

The stock solution of the *Abies sibirica* L. was prepared by dissolving 1 mL of polyprenol sample in the solvent mixture (35% isopropanol/65% methanol) at room temperature (r.t.). The solution was then mixed on a Vortex (YellowLine TTS 2, serial num. 03.169241) for 1 min at 2500 rpm. The resulting solution was filtered through a 0.45 mm MS Nylon Syringe filter. Separate solutions were used to prepare the calibration standards. The sample solution was centrifuged using Mikro 200R (Hettich Zentrifugen, Serial Num. D – 78532), and the supernatant was directly injected into the HPLC-DAD-MS system. For polyprenol isolate identification purposes, a reference standard mixture solution (PP mix of P14-P18; Batch 08-10 JTC-*Pinus sylvestris* L.) was used, and additional peak identification using HRMS data was carried out.

### 4.4. Reverse-Phase UHPLC Analysis

UHPLC analyses were performed using an Agilent 1290 Infinity series (Agilent Technologies Deutschland GmbH, Waldbronn, Germany) UHPLC equipped with an Agilent 1290 photodiode array detector (DAD). A C18 reversed-phase packing column (Extend C18 2.1 × 50 mm; 1.7 μm; Agilent Technologies Inc., Wood Dale, IL, USA) was employed for the separation. The column was thermostatted at 40 ± 1 °C. The qualitative analyses were achieved at a wavelength of 210 nm. The injection volume was 0.5 µL. The mobile phase was directly on-line degassed, and its composition consisted of isopropanol (CH_3_)_2_CHOH (Merck, Branchburg, NJ, USA, HPLC grade) (Solvent A) and methanol CH_3_OH (Fisher Scientific UK, Loughborough, United kingdom, HPLC grade) (Solvent B) at a flow rate of 0.22 mL/min with following elution program: initial – 35% A, 65% B for 1.5 min; 75% A, 25% B 1.5–10 min, constant amount of 75% A, 25% B was kept for 15 min, 45% A, 55% B 25–30 min, finally 35% A, 65% B 30–32 min [53].

### 4.5. Identification of Polyprenols by TOF LC/MS

The source parameters for high-resolution time-of-flight mass spectrometry were: drying gas flow 10 mL/min at 325 °C. Ion fragmentation was achieved at ionization voltage of 85 V at the detection range *m*/*z* 100–2000 using internal precision mass calibration solution at *m*/*z* 121.050873 and *m*/*z* 922.009798 (G1969-85001 ES-TOF Reference Mass Solution Kit, Agilent Technologies & Supelco Inc., Munich, Germany). Quantities of separated PP were analyzed by external quantitative standard Pren-14 (Poland) and a mixture of polyprenols (Standard P14-P18 mix *Pinus sylvestris* L. Batch 08-10 JTC; Code 89-5170, Poland). The calibration curve of the standard solution was constructed by plotting the ratio of the average chromatographic peak area and mass concentration of the Pren-14, obtained using DAD detection. According to the reflected data, the regression equation of the trend line was calculated. Standard solutions were injected in triplicate, and the corresponding peak areas were recorded. The relative standard deviation was determined to be less than 2.0%. The obtained calibration curve showed a linearity of the correlation coefficient (R2) in the concentration range of 0.9978. The coefficient of determination (R2) was calculated using Microsoft Excel 2013, *p* < 0.001.

### 4.6. Preparation of Proliposomal Polyprenol Solution 1:17 w/w

4.7g of the Lipoid Phosal 40IP was placed in the 10 mL glass vial. The lipid mass was mixed using a magnetic stirrer, and 3 g of polyprenols were added in small portions and mixed with the Lipoid Phosal 40IP lecithin until the uniform solution was achieved.

### 4.7. Determination of Solubility of Polyprenols in Ethanol and Ethanolic Solution of Lecithin

The aim of this experiment was to determine how lecithin content in ethanol solution can modulate the solubility of polyprenols. To the 100 mg of polyprenols in the 5 mL glass test tubes, 2.0 mL of ethanol solutions of soya phosphatidylcholine (Lipoid P75) (0%–25%) was added until no further solubility was achieved after extensive vortexing over two minutes at 24 °C. In the case of 15% phosphatidylcholine solution in ethanol, higher polyprenols dose was required to achieve saturation. For 25% phosphatidylcholine solution no saturation was achieved and polyprenols could be mixed at every proportion with 25% lecithin in ethanol. The solutions were left for stabilization for 1 h (with occasional mixing), and afterward samples were centrifuged in order to spin down the polyprenol droplets in the 1.5 mL Eppendorf tubes at 14 000 rpm (5 min.). The transparent supernatants were then taken and diluted in chloroform in order to determine the polyprenol content by the HPLC method. The solubility of polyprenols in the 25% lecithin solution was not assessed by any instrumental method.

### 4.8. Determination of Incorporation Efficiency of the Polyprenols in Liposomes

In order to determine how polyprenols/lipid ratio affects the incorporation efficiency of the polyprenols in the liposomes composed from two commercially available fluid lecithin products, polyprenols were mixed with fluid Lipoid Phosal 40 IP lecithin and Lipoid Phosal 75 SA lecithin at the polyprenol ratio ranging from 1:5 to 1:40 *w*/*w*. Polyprenols are liquids, therefore free substances are difficult to remove from liposomal samples, since polyprenols, being practically insoluble in water, cannot be separated from liposomes by dialysis or another similar method. Thus, to determine the amount of polyprenols incorporated within multilamellar liposomes (MLVs), a method based on the difference of the density of liposomes and polyprenol droplets was established. 100 mg of the polyprenol/lecithin mixtures (1:5 to 1:40 *w*/*w*) were placed in the glass test tubes, and then 900 μL of water was added to the tubes. The mixture was mixed over 30 sec. using vortex (IKA, Breinsgau, Germany) to achieve liposomal suspension (or polyprenol droplets). From this suspension 100 µl samples were collected and mixed with 900 µl of 200 mM sucrose solution in the 1.5 mL Eppendorf tubes. Then the samples were centrifuged 10 min. at 14,000 rpm in order to separate liposomes with polyprenols from polyprenol emulsion. The upper (floating) polyprenol oily fraction was removed and the phospholipids and polyprenols were analyzed in the MLV containing supernatants. The phospholipids’ content was determined using the modified Stewart assay, and the polyprenol content was assessed using the HPLC method. The achieved ratio was compared with the initial values (not centrifuged samples) in order to calculate the polyprenol incorporation efficiency.

### 4.9. The HPLC Determination of Polyprenols in Liposomes

Polyprenol concentration was determined by the HPLC method. Briefly, lipids or lipid solutions were diluted in chloroform. In the case of liposomes, those were first dissolved in isopropanol (1:10) and then diluted in chloroform. The polyprenol concentration was measured with an HPLC system Waters 660 Pump, Nova-Pak^®^Silica (150 mm × 3.9 mm, 5 μm) column, and chloroform or chloroform:hexane (90:10, *v*/*v*) mobile phase with a flow rate of 1 mL/min. The detection was done using a Waters 996 photodiode array detector at 335 nm (Waters corp., Milford, Mass., USA).

### 4.10. Preparation of Polyprenol Liposomes for Light and Electron Microscopy

For light microscopy, the proliposomal lipid mass containing polyprenols was mixed with water (5% lipid concentration) and vortexed vigorously to achieve homogenous liposomal suspension. Two approaches were used to stain the liposomes. In method A: 100μL of liposomes were mixed with the same volume of 2% ammonium molybdate, and then the mixture was placed on a Formvar copper grid and dried at room temperature. In method B: the liposomal specimens were placed on the Formvar copper grid and dried out at the room temperature for 15 min. After that, a small droplet of the 2% ammonium molybdate solution was placed onto the grid and the excess of the solution was blotted with filter paper. Prior to the microscopic observation, both grids were dried out at the same conditions as mentioned above. Method A gave positive staining, whereas method B gave negative staining.

### 4.11. Size Distribution of Liposomes containing Polyprenols

Before measuring the polyprenol loaded liposomes were diluted in water, and then the mean diameter size (volume weighting) and power-distance index (PDI)were assessed. The determination was performed at room temperature (25 °C) using a Zetasizer Nano ZS (Malvern Instruments, Malvern, UK).

## 5. Conclusions

Polyprenols from Siberian fir lipophilic extract were identified via separation of their polyprenol homologs. The quantified Abies *sibirica* L. polyprenol chain lengths were predominantly C55–C100, which corresponded with previous literature findings. The purified 80% polyprenols were efficiently incorporated into soybean phosphatidylcholine multilamellar liposomes at a ratio of 1:17. A new method based upon co-solubilization and modified ethanol injection method of polyprenols in the ethanol-phospholipid system was developed. Due to multiple known effects of the natural conifer isoprenoids — antioxidative, anti-inflammatory, hepatoprotective, etc. *Abies sibirica* L. polyprenols might be appropriate candidates for food supplements, cosmetic formulations and in synergistic combinations with other therapies against various health disorders, such as statin-induced myopathies, Alzheimer’s disease, multiple sclerosis, non-alcoholic fatty liver disease, and alcohol-induced liver cirrhosis.

## Figures and Tables

**Table 1:** Identification and quantification of the separated polyprenol homologs from Siberian fir (*Abies sibirica* L.).

Polyprenol	Molecular Formula	t_R_, min (UHPLC-DAD)	Relative Amount, % *Abies sibirica* L.	Mode of Identification	[M + Na]^+^ (HRMS)
P11	C_55_H_90_O	4.86	0.06 ± 0.01	Standard/HRMS	789.6867
P12	C_60_H_98_O	5.88	0.26 ± 0.04	Standard/HRMS	857.7503
P13	C_65_H_106_O	7.16	2.05 ± 0.04	Standard/HRMS	925.8134
P14	C_70_H_114_O	8.78	15.26 ± 0.78	Standard/HRMS	993.8779
P15	C_75_H_122_O	10.81	37.23 ± 0.56	Standard/HRMS	1061.9372
P16	C_80_H_130_O	13.25	29.11 ± 0.51	Standard/HRMS	1130.0017
P17	C_85_H_138_O	14.86	11.31 ± 0.01	Standard/HRMS	1198.0650
P18	C_90_H_146_O	15.98	3.36 ± 0.06	Standard/HRMS	1266.1274
P19	C_95_H_154_O	16.86	1.01 ± 0.04	Standard/HRMS	1334.1893
P20	C_100_H_162_O	17.59	0.31 ± 0.11	Standard/HRMS	1402.2577

**Table 2:** Liposomal polyprenol sample compositions and appearance.

Lipoid/lecithin Amount	Ethanol Amount	PolyprenolAmount	Water Amount	Appearance of the Sample	Stability after One Month	Liposome Structure
Lipoid P45/150mg	50mg	30mg	up to 5g	Semiliquid	Not stable	Oligolamellar
Lipoid P45/150mg	50mg	30mg	up to 5g	Semiliquid	Not stable	Oligolamellar
Lipoid P75/105mg	50 mg	30mg	up to 5g	Semiliquid	Not stable	Oligolamellar
Lipoid P75/150 mg	50mg	30mg	up to 5g	Semiliquid	Not stable	Oligolamellar
Lipoid Phosal 40 IP/1000mg	0mg	30mg	up to 5g	Liquid	Stable	Multilamellar
Lipoid P45/500mg	150mg	30mg	up to 5g	Semiliquid	Stable	Oligolamellar
Lipoid P45/1000mg	200mg	30mg	up to 5g	Dense jelly paste	Stable	Uni/Oligolamellar

## Supplementary Materials

The following are available online at https://www.mdpi.com/1420-3049/25/8/1801/s1, Figures S1–S10: High-resolution mass spectra (HRMS) examples of polyprenol homologs P11–P20.
