# Supplementary material for: Identification of Abies sibirica L. Polyprenols and Characterisation of Polyprenol-Containing Liposomes
Source: Molecules. 2020 Apr 14;25(8):1801. doi: 10.3390/molecules25081801 (PMC7221546; doi:10.3390/molecules25081801)
Supplement: Supplementary file 1 [file molecules-25-01801-s001.pdf]

Supplement 1

As a reference of the studied polyprenol homologs below we provide high-resolution mass spectrometry (HRMS) peaks for *Abies sibirica* L. polyprenols.

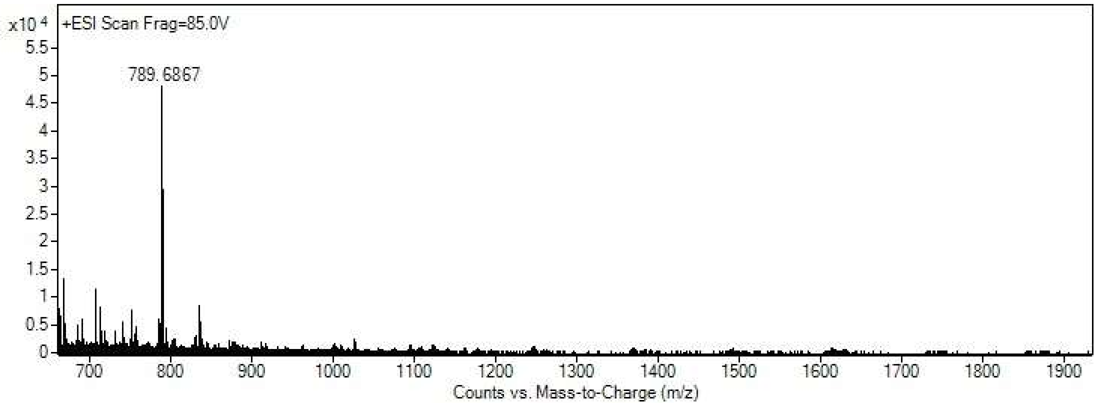

Figure S1. High-resolution mass spectra (HRMS) example of polyprenol homologue P11.

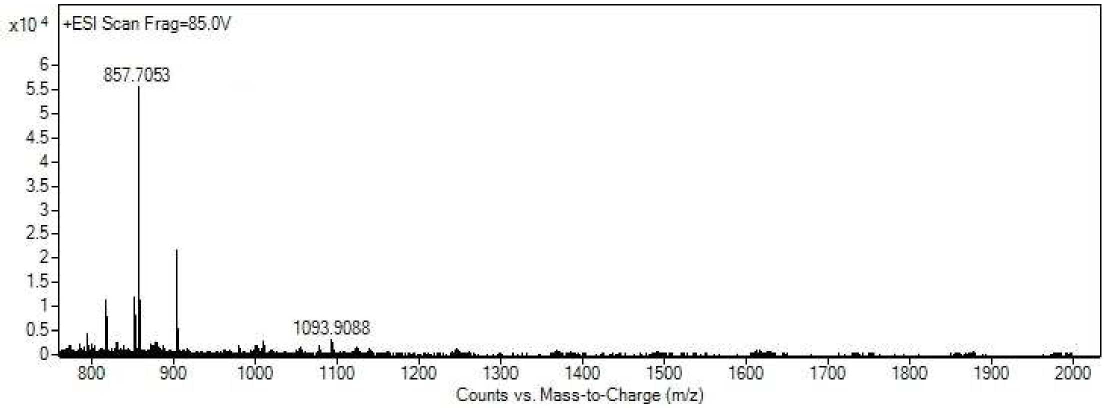

Figure S2. High-resolution mass spectra (HRMS) example of polyprenol homologue P12.

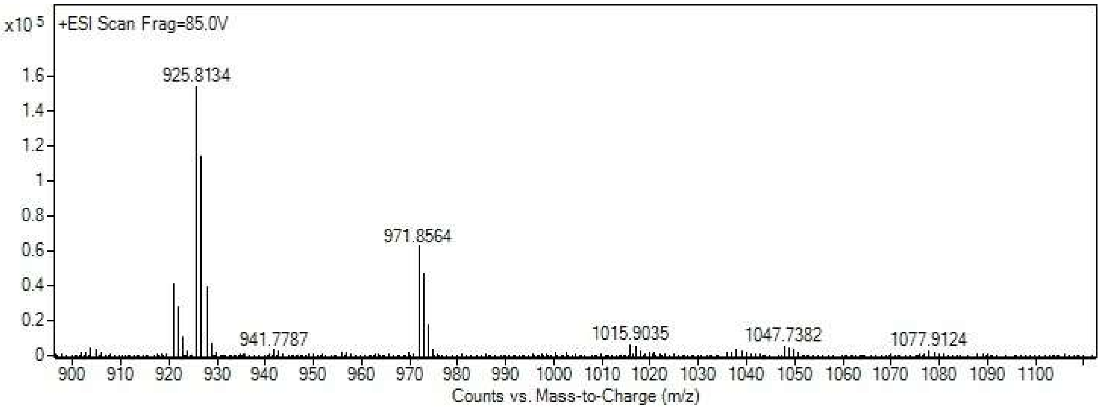

Figure S3. High-resolution mass spectra (HRMS) example of polyprenol homologue P13.

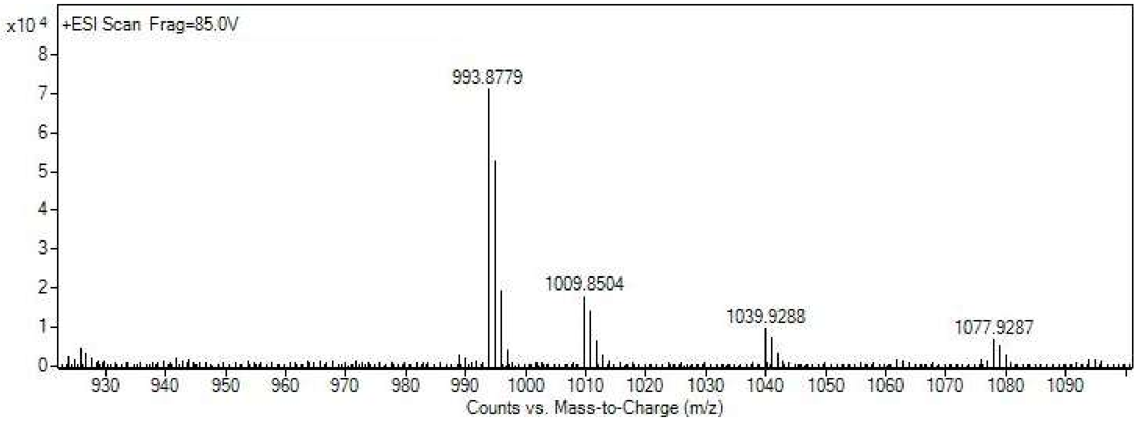

Figure S4. High-resolution mass spectra (HRMS) example of polyprenol homologue P14.

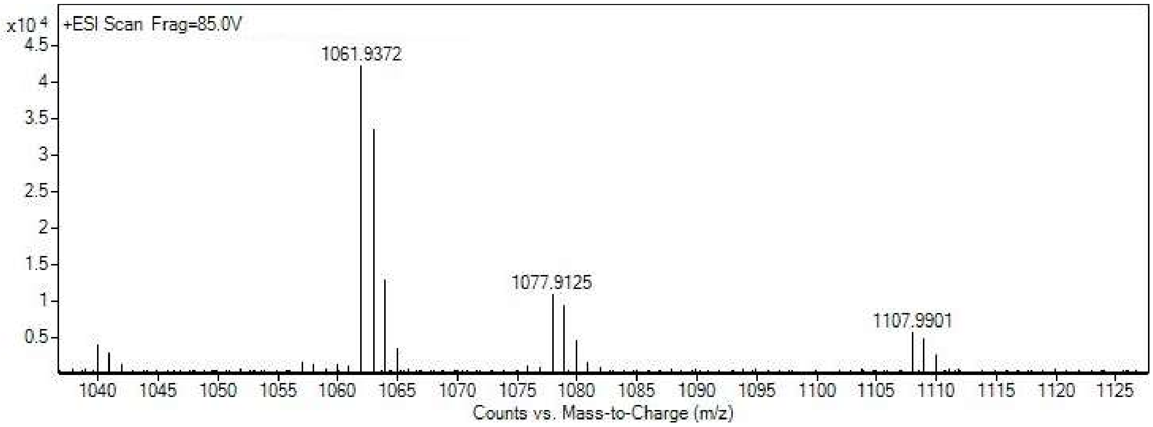

Figure S5. High-resolution mass spectra (HRMS) example of polyprenol homologue P15.

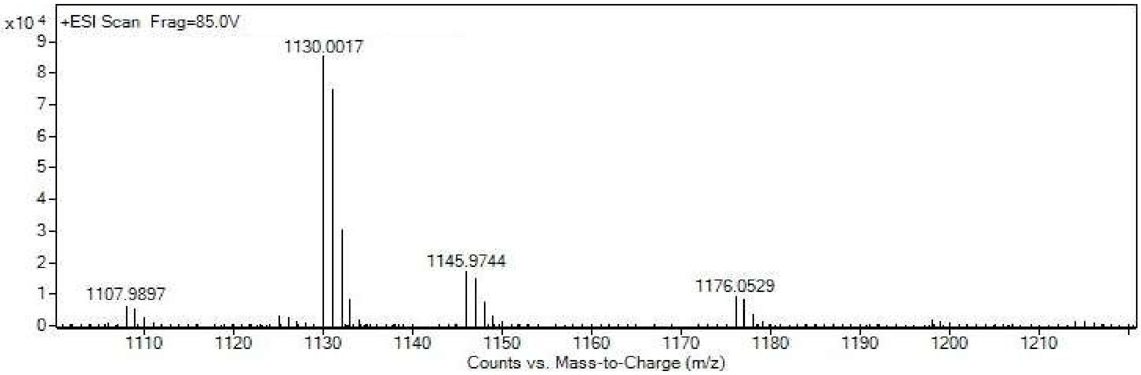

Figure S6. High-resolution mass spectra (HRMS) example of polyprenol homologue P16.

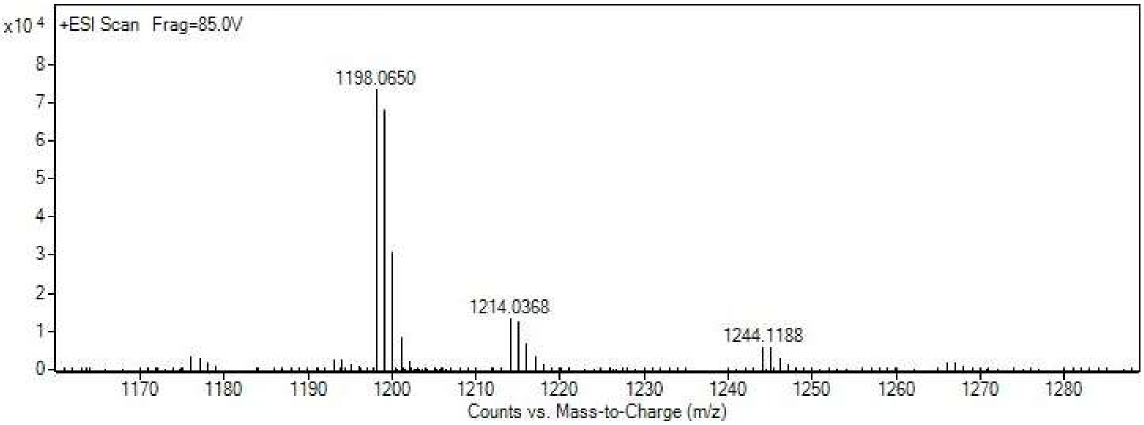

Figure S7. High-resolution mass spectra (HRMS) example of polyprenol homologue P17.

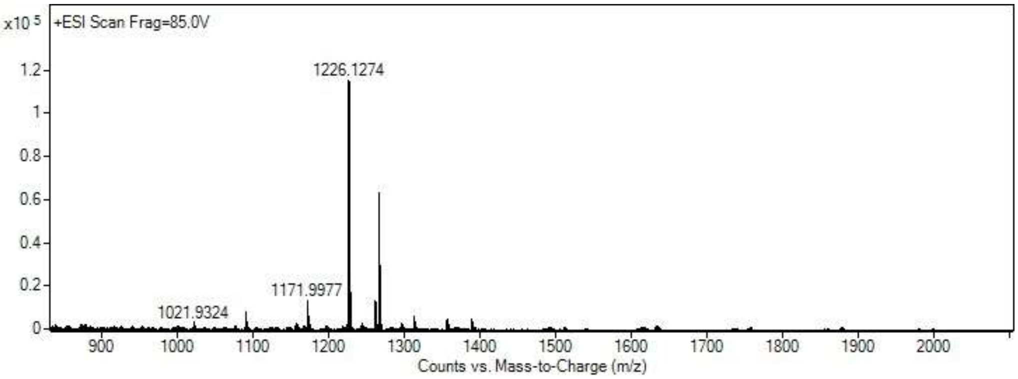

Figure S8. High-resolution mass spectra (HRMS) example of polyprenol homologue P18.

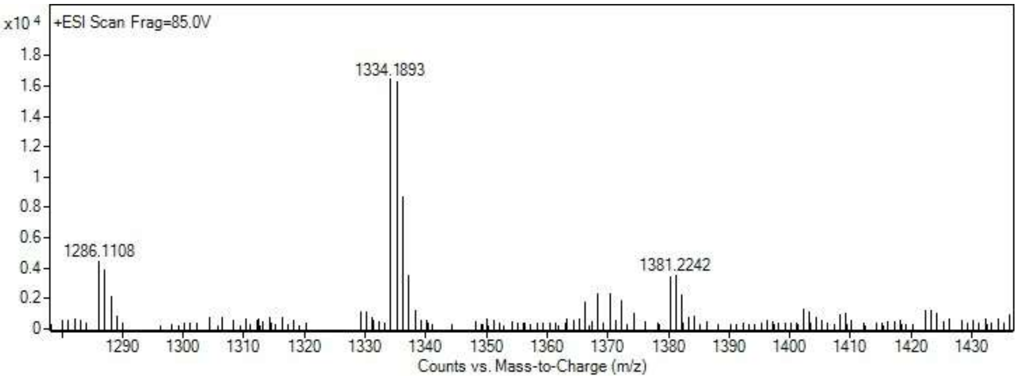

Figure S9. High-resolution mass spectra (HRMS) example of polyprenol homologue P19.

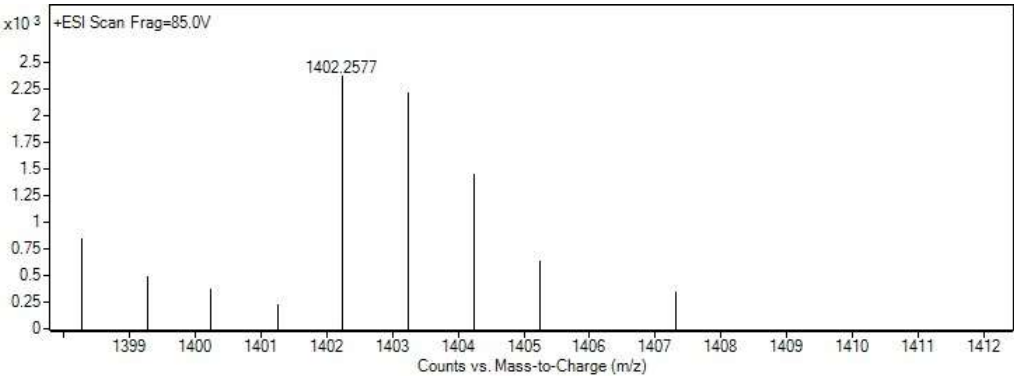

Figure S10. High-resolution mass spectra (HRMS) example of polyprenol homologue P20.
